# In Vitro Determination of Inhibitory Effects of Humic Substances Complexing Zn and Se on SARS-CoV-2 Virus Replication

**DOI:** 10.3390/foods11050694

**Published:** 2022-02-26

**Authors:** Polett Hajdrik, Bernadett Pályi, Zoltán Kis, Noémi Kovács, Dániel Sándor Veres, Krisztián Szigeti, Ferenc Budán, Imre Hegedüs, Tibor Kovács, Ralf Bergmann, Domokos Máthé

**Affiliations:** 1 Department of Biophysics and Radiation Biology, Semmelweis University, Üllői út 26., H-1085 Budapest, Hungary; h.polett0809@gmail.com (P.H.); kovacsnoi@hotmail.com (N.K.); veres.daniel@med.semmelweis-univ.hu (D.S.V.); krisztian.szigeti@gmail.com (K.S.); hegedus.imre1@med.semmelweis-univ.hu (I.H.); rkbergmann@web.de (R.B.); 2 National Biosafety Laboratory, National Public Health Center, Albert Flórián út 2-6, H-1097 Budapest, Hungary; palyi.bernadett@nnk.gov.hu (B.P.); kis.zoltan@nnk.gov.hu (Z.K.); 3 Department of Microbiology, Semmelweis University, Üllői út 26., H-1085 Budapest, Hungary; 4 CROmed Translational Research Ltd., Tűzoltó u. 37-47, H-1094 Budapest, Hungary; 5 Institute of Transdisciplinary Discoveries, Medical School, University of Pécs, Ifjúság útja 11, H-7624 Pécs, Hungary; budfer2@gmail.com; 6 Institute of Physiology, Medical School, University of Pécs, Szigeti út 12, H-7624 Pécs, Hungary; 7 Institute of Radiochemistry and Radioecology, University of Pannonia, Egyetem u. 10., H-8200 Veszprem, Hungary; kovacstiborjanos@gmail.com; 8 Helmholtz-Zentrum Dresden Rossendorf, Bautzner Landstraße 400, 01328 Dresden, Germany; 9 Hungarian Centre of Excellence for Molecular Medicine, In Vivo Imaging Advanced Core Facility, Semmelweis University Site, Római Blvd. 21, H-6723 Szeged, Hungary

**Keywords:** SARS-CoV-2, humic acid, fulvic acid, Zn-Se-ascorbic acid complex, antiviral activity, RT-PCR

## Abstract

(1) Background: Humic substances are well-known human nutritional supplement materials and they play an important performance-enhancing role as animal feed additives. For decades, ingredients of humic substances have been proven to carry potent antiviral effects against different viruses. (2) Methods: Here, the antiviral activity of a humic substance containing ascorbic acid, Se^−^ and Zn^2+^ ions intended as a nutritional supplement material was investigated against SARS-CoV-2 virus B1.1.7 Variant of Concern (“Alpha Variant”) in a VeroE6 cell line. (3) Results: This combination has a significant in vitro antiviral effect at a very low concentration range of its intended active ingredients. (4) Conclusions: Even picomolar concentration ranges of humic substances, Vitamin C and Zn/Se ions in the given composition, were enough to achieve 50% viral replication inhibition in the applied SARS-CoV-2 virus inhibition test.

## 1. Introduction

**Figure 1:**
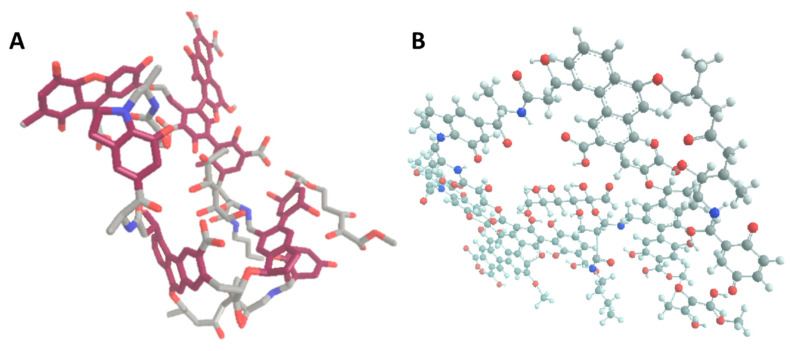
**Panel A** Energy optimized molecular structure of a small, characteristic part of humic substances (HSs). The optimal 3D structure was calculated by energy minimization of the molecule. This graphic contains typical structural elements and bonds of natural HS that randomly build up their molecular structure, based on NMR and IR studies [7]. **Panel B**: Atom model of the possible structure of a humic acid monomer. Grey, carbon; white, hydrogen; blue, nitrogen; red, oxygen atoms. Claret colour discerns the carbon atoms belonging to the characteristic polyphenolic aromatic rings (Graphics and energy optimization calculation was created using ChemDraw Ultra and Chem3D software).

Humic substances (HSs), composed by hymatomelanic acid, humic acids (HAs), fulvic acid (FA), ulmic acid, and trace minerals are widely known basic components of nutritional supplement materials in humans. A large quantity of HSs are generated in forests and peat. HSs have a ubiquitous presence and HSs are one of the largest carbon sources in nature. HSs originate from decayed plants in the soil that are decomposed by microbes. HSs are soluble in alkaline media, partially soluble in water and insoluble in acidic media. The chemical composition and physiological effects of HSs have been investigated for at least the last 90 years [1,2]. The chemical building blocks of HSs are mainly carboxylic acids, phenolic and alcoholic hydroxyl groups, quinonoid and aliphatic carboxyl groups, and methoxy groups [3,4,5] (Figure 1). The exact composition of HSs may differ based on their origin and type of extraction technology. Humic substances show various physiological effects on living organisms, e.g., they have hormone-like effects, regulate genes, activate different signal transduction processes through interactions with membranes, regulate ion-exchange, and modulate stress-reactions, etc. [4]. HSs are used as components of fungotherapy or mud therapy and their beneficial features are well-known [6], but the correlation between the physiological effects and the chemical compounds or the relevant biochemical pathways are not clear yet. 

One of their most important components, FA, is water-soluble thanks to its polyphenolic molecular structure (Figure 1). Water molecules stabilize their electronic structure even in neutral form [8]. Fulvic acid forms small aggregates at pH 5 on gold surfaces and, presumably, these aggregates also form in soil microstructures. These aggregates compose a fractal-like structure at pH 8 caused by electrostatic forces, according to surface-enhanced Raman Spectroscopy measurements (SERS) [9]. The average molar mass of FA is highly dependent on its origin. Native FA is usually a mixture of different molecules [10]. The molecular weight of native FA (e.g., originating from the Suwannee River) is about 2310 Da as provided by literature and the International Humic Substances Society (IHSS) [11,12]. The other important and well-studied component, HAs, is larger and more complex in structure with much less solubility. The molecular weight of HAs changes from 3160 to 26,400 Da depending on the origin of the samples [13]. Contrary to the molecular weight differences, no significant difference is detectable between the structural building block elements of FA and HAs [3,14,15,16]. 

HSs have been used for thousands of years in human healthcare. Humic substances are well-known drugs in Indian Ayurvedic medicine as “shilajit” [17,18,19,20] since time immemorial. They were also used in European mud- and balneotherapy from ancient times [21]. Shilajit contains mainly FA, HAs, and trace minerals. Numerous studies show that HSs have anti-inflammatory, antioxidant, antimutagenic, antiviral, heavy metal chelating, antitumour, pro-apoptotic, and photo-protective properties [19]. The first formal scientific reports on HS as therapeutic agents in modern Western clinical medicine are from 1957 in Hungary [22,23]. Chinese medical literature also abounds with the therapeutic application of HS, which have constituted an important therapeutic class in Traditional Chinese Medicine for over 3000 years. Humic substances have since been clinically tested and found to confer numerous beneficial features, such as potentially protecting the human body against blood coagulation or fibrinolysis or decreasing the effects of ionizing radiation [24].

Humic acids (HAs) have good antioxidant activity and free radical scavenger ability [25]. 

HSs seem to have very few adverse effects (and even those only in specially hindered nutritional circumstances) and they can be given as food and feed supplements [26]. In addition to their use in food, HS have wide agricultural and environmental uses, e.g., as plant nutrients [27]. Currently, humic matter is not only a human nutritional supplement, but it forms the basis of numerous feed additives to improve animal growth performance and health, even by replacing antibiotic performance enhancers [28]. HS also have effects on gastrointestinal ulcers in pigs [29] or rats [30]. HAs forms a protective film on the surface of the ulcer in the stomach and helps with cellular regeneration [29]. Fulvic acid (FA), combined with probiotics, enhances the digestibility of phosphorous and metals. Moreover, it increased immune capability in a study conducted on a convincingly large number of pigs [31]. HSs as nutritional supplements have numerous beneficial effects on microelement and trace element homeostasis, e.g., on iron and manganese homeostasis as proven in a rat model [28], as well as copper and zinc homeostasis [32]. Some compounds of HS were found to have neuroprotective effects in animal studies, e.g., anthocyanin-containing gold-FA coated nanoparticles could prevent Alzheimer’s disease signs in rats [33]. FAs not only reduce the assembly of tau proteins but also restore the original tau folding state [34]. Some recent reports have concluded that numerous compounds of HSs, e.g., fulvic acid [35], are promising candidates for pharmaceutical use to enhance drug delivery of active pharmaceutical ingredients (APIs) [25,36]. HSs not only have additional effects on health, but they also have some therapeutic potential [25,37,38]. Fulvic acid has a clinical beneficial effect in chronic inflammatory diseases and diabetes [39]. The full HSs fraction also has demonstrated anti-inflammatory activity [40,41,42].

HSs have been known as antiviral agents for decades. Not long ago, a drink based on HS and plant extracts, branded as “Secomet V” was reported to possess broad spectral antiviral properties, e.g., as anti-HIV, anti-poxvirus, and anti-SARS activities [43]. Numerous studies have investigated and then reported antiviral features of HSs, e.g., against Coxsackie virus A9 [44], Herpes Simplex virus Type I. [45,46,47], Influenza virus A/WSN/1933 (H1N1) [48], or HSs operating as immune stimulators in Human Immunodeficiency virus-1 (HIV-1)-positive patients [49,50,51], and tick-borne encephalitis virus infection (TBEV) [52]. A very recent high-quality study found high antiviral activity of HSs compounds against HIV-1 [53]. Moreover, FA can destroy different strains of a highly mutating H5N1 influenza virus [54]. This fact suggests that HSs can play a leading role in development of “broad spectrum” antiviral agents [54]. They could also provide beneficial effects as prophylaxis and therapy of coronaviral diseases [55]. A recent study shows that FA and iodine complex reduces significantly the virulence of SARS CoV-2 virus in a Vero 76 cell culture [56]. Natural HSs samples as polyelectrolytes containing numerous physiologically active elements (Fe, P, S, Si, K, Ca, Mn, Cu, Zn) as complexes, also significantly reduce viral infection of SARS CoV-2 in Vero E6 cell line [57]. HS could also be regarded as pre-biotics in the gut, playing a broad role in modulating the immune system. 

Given the historical records and the scientific knowledge about the undisputedly high activity of HSs against human viruses for more than 50 years, the authors attempted to investigate the possible antiviral activity of a commercially available nutritional supplement material. An in vitro quantitative real time Polymerase Chain Reaction (RT-qPCR)-based viral replication inhibition test was applied, using different dilutions of the investigated HSs material. 

In this material peat extract, HSs are enriched with Zn^2+^ ions, Se^−^ ions, and ascorbic acid. Numerous studies confirmed that both ascorbic acid [58,59], selenium ions [60,61], and zinc ions [58,62,63], one by one, have anti-inflammatory effect and could reduce symptoms of COVID-19 infections [64]. HS effectively bind metal ions as chelates [65,66,67]. Aromatic molecules and groups, e.g., pyrene or Zn^2+^ ions, Se^−^ ions and ascorbic acid containing HSs macromolecules could function with a nanoparticle-like colloidal behaviour [68], which can enhance the antiviral activity of drug molecules [69] by virtue of their high surface to mass ratio. Our goal was to experimentally investigate how quantitatively the tested food supplement decreases in solution the amount of SARS-CoV-2 viral gene copy numbers detectable by qPCR in an established Vero E6 cell culture model of infection.

## 2. Materials and Methods

The Test Article (TA) is a nutritional supplement base material available commercially from its producer (ZnSeC-Humicin, Humic2000 Ltd., Budapest, Hungary) in a powder or as a solution and was commercially sourced by the authors. The purchased TA was sent to an independent testing laboratory (Balint Analitika Ltd., Budapest, Hungary) for the determination of ingredient concentrations using standardised, quality-assured and certified analytical test methods as prescribed in the Codex Alimentarius Hungaricus (Hungarian Food Book). All further in vitro cytopathogenicity and antiviral activity tests were performed at the Biosafety Level 3 (BSL-3) National Biosafety Laboratory (Budapest, Hungary). 

### 2.1. Preparation of Dilutions

The stock solution applied in our study was 10 g of ZnSeC-Humicin powder dissolved in 1000 mL of Dulbecco’s Modified Eagle Medium (DMEM, Gibco/ThermoFisher, Waltham, MA, USA) cell culture medium. All further dilutions were prepared using DMEM.

Final dilutions were 100-fold, 500-fold, 1000-fold, 2000-fold, 5000-fold, and 7000-fold of the original test article.

### 2.2. Cell Culture

VeroE6 cell line (ATCC^®^ CRL-1586™) was maintained in DMEM with the addition of 10% m/m Fetal Bovine Serum (FBS, Gibco/ThermoFisher, Waltham, MA, USA) and 50 U/mL penicillin and 5 µL/mL streptomycin. Then, 2 × 10^5^ cells/well VeroE6 cells were seeded to 96-well culture plates, grown to monolayer, and used for the viral inhibition assay protocol. Cytopathogenicity of the stock solution in vitro was tested by adding 200 µL of TA stock solution to each well with growing, uninfected VeroE6 cells and incubating for 48 h. 

### 2.3. Test for the Inhibition of SARS-CoV-2 Replication

To detect viral replication, we examined the supernatants from VeroE6 cells cultured for 48 h in the presence of the TA dilutions. The assay was performed using qRT-PCR and the QuantiNova Probe RT-PCR kit (Qiagen, Düsseldorf, Germany), and the LightCycler 480 Real-Time PCR System (Roche, Basel, Switzerland). RNA extraction was performed using the Roche High Pure Viral RNA Kit (Roche, Basel, Switzerland) according to the manufacturer’s instructions. 

We then targeted the N-(nucleoprotein) gene of SARS-CoV-2 virus with Roche Lightcycler 480 RNA Master Hydrolysis Probes (Roche Basel, Switzerland) with primers developed in-house, using the Lightcycler 2.0 (Roche) platform.

For the cytotoxicity assay a spectrophotometer (Omni cell adhesion light spectrometer, Cytosmart, Netherlands) was used. The percentage rate of surface covered by monolayers and of transparent surface (plaques appearing in the absence of cells killed by the virus) in all the wells of the 96-well cell culture platform were determined. 

All tests were made in triplicates. From these, the relative parameters of viral inhibition were calculated compared to the viral effect in an infected, untreated cell culture free of TA. 

### 2.4. Test Protocol

Different experimental groups were applied: the “virus control” group was a positive control, untreated with any TA, to determine the effects of the virus infection. The inhibitory effect of TA dilutions was compared to the result of this group.

The cell control group was a negative control, where the effect of just the culturing activities on virus-free cells was measured; the group was also free of the TAs.

Infection test groups were set up with each TA dilution added to the wells and incubated with the virus as follows. In the infection phase, the TA dilutions were mixed in a 1:1 volumetric ratio with 100 TCID50 SARS-CoV-2 virus suspension. This mixture was put in the cell culture and then incubated at 37 °C in 5% CO_2_ atmosphere for 2 h in the absence of FBS.

After the infection phase, the infectious virus-containing mixture was removed with an automatic pipette. For the incubation phase, immediately, a 1:1 mixture of VP-SFM medium (Gibco/ThermoFisher, Waltham, MA, USA) and the appropriate DMEM medium dilution of the TA was added to the wells in equally 200 microliter volumes. Incubation with this TA-treated medium lasted for 48 h. Thus, the TA was in the culture both for the primary infection and for the subsequent 48 h of incubation.

After 48 h, the virus-containing medium (with virions produced in the cell culture after the primary challenge-virus suspension was removed) containing the TA was removed from the cells. The removed viral supernatant was ultracentrifuged at 16,.000 g and the supernatant was further measured for viral RNA content.

The supernatant RNA contents were quantified for gene copy numbers and compared to the number of copies measured in the untreated, not inhibited SARS-CoV-2 infected cell cultures to calculate the inhibitory effect.

### 2.5. Calculations

Viral N-gene copy numbers obtained in the triplicates both for inhibitory TA and for untreated virus control wells were used as input data. 

A sigmoidal growth function was generalised as (Equation (1))

(1) $$F\left(x\right)=\frac{M}{1+e^{-b\left(x-a\right)}},$$

where *M* as a constant is the maximal viral copy number (at infinite dilution of the added TA, i.e., with only infinitesimally small TA content of the plate wells); *a* is the dilution value of the TA at 50% of M (Dilution50); *b* is the derivate of intermediate transmission.

One then characterised the curve fit and calculated the value of parameter *a* and its 95% confidence interval. As a further step towards obtaining ID_50_ values, the ingredient molar concentrations were determined from the obtained Dilution50 (“*a*”) value using the known composition, the applied volume per well, and molar masses of the TA ingredients. 

## 3. Results

Neither qualitative nor quantitative cytotoxicity was observed in any of the dilutions of the tested humic substance complex on VeroE6 cells. Moreover, a dose-dependent inhibition of SARS-CoV-2 replication was demonstrated in the same cell line with SARS-CoV-2 virus B1.1.7 VOC as infective agent.

The independent laboratory measurements revealed that original HS components (65.9 m/m% FA and 0.5 m/m% HA) are compounded with high concentration of Vitamin C (about 40 g/100 g) and lower-concentration of metal ions (60,900 mg/kg Zinc ion and 23.2 mg/kg Selenium ion) according to their recommended daily intake values for humans (Table 1).

The obtained dilution value and other inhibition curve characteristics are presented in Table 2. 

**Figure 2:**
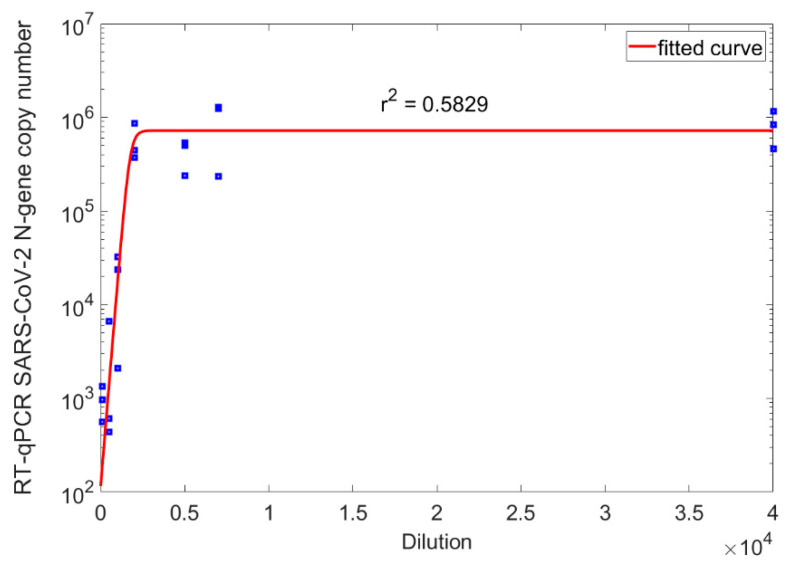
Inhibitory effect of humic substances, compounded with Zn-Se-ascorbic acid complex for SARS-CoV-2 virus B1.1.7 variant of concern (“Alpha VOC”) in Vero E6 cell culture.

Test results here reported show that the humic substances compounded with Zn^2+^-Se^−^-ascorbic acid (ZnSeC-Humicin) seem to exert a significant inhibitory effect on SARS-COV-2 virus replication (Figure 2). A sigmoidal curve (red continuous line) could be fitted to the triplicate copy number data points (blue squares) for determination of the mean inhibitory dose (ID_50_) value of the tested HS material. Figure 2 shows a semi-logarithmic scale transformation of the sigmoidal function. It was found that the Dilution50 value is 1740-fold of the applied ZnSeC-Humicin stock.

Molar concentrations of ingredients of ZnSeC-Humicin at dilution in the ID50 value are usually in a 10^−9^ M (pM) concentration range. The measured relative humidity of the powder was 8.5% m/m thus data were corrected to dry weight in the table. A very low, 70.80 picomol/L concentration of FA and a similarly also very low, femtomolar (49.00–409.00 fM) concentration of HA with 610.00 pM ascorbic acid, 251.00 pM Zn^2+^, and 7.28 pM of Se^−^ can reduce the copy numbers of the detected SARS-CoV-2 virus genes (“Alpha VOC”) B1.1.7 variant of concern by half (Table 3). 

Other studies in the literature have also used HS or FA as therapeutic element carrying agents in cell culture against SARS-CoV-2. Uspenskaya et al. found that the half maximal inhibitory concentration (IC_50_) of natural HS is 0.023–0.041 mg/mL during the treatment of Vero E6 cell culture against SARS-CoV-2 virus [57]. Köntös discovered that 200 μg/mL concentration of iodine-fulvic acid clathrate complex reduced the infectious dose of SARS-CoV-2 to 1% of its original value using Vero 76 cell culture [56]. These results are comparable to those here presented. The low effective concentrations of HS or FA suggest that combining HS with additional elements and ions could result in effective HS-ion complexes against the SARS-CoV-2 virus.

## 4. Discussion

Despite its successful therapeutic usage over thousands of years, experimental knowledge about the molecular mechanisms of HSs biological effects remains scarce. In previous decades, many scientific results have robustly established the general antiviral qualities of humic substances. This investigation is rooted in those precedent scientific studies stretching over several decades.

The antiviral activity of HSs molecules depends on the presence of different acidic functional groups, e.g., carboxylic groups in the HS sample [45]. The existence of these acidic groups, mainly aliphatic and aromatic carboxylic groups and *p*-diphenyl groups and their numerous methylated, etc., derivatives, can highly increase the antiviral effectivity of HSs samples, e.g., against HIV virus [46]. Furthermore, the water-soluble fraction of native HS (oxihumate) reduces the cell binding of HIV virus by interference with CD4 binding and V3-loop mediated entry of virus to infected cells [50]. Additionally, HS have an activating effect on the immune system, e.g., HS increases twofold the IL-2 secretion of mouse splenocytes [38].

All three compounds (HS, Selenium(−) ion and Zinc(2+) ion) have antiviral activity against SARS-CoV-2 alone, while the mixture of these compounds can act synergistically. Ionophore-bound Zinc ions have been experimentally shown to exert a strong anti-coronaviral effect against SARS virus by te Velthuis et al. in 2010 [63]. Humic acids act as ionophores for Zinc ions, as demonstrated by published works of Smirnova [70] and Krezel [71]. The nutritional role of Zinc(2+) ions in the fight against many coronaviruses has also been recently published [72]. As shown by Zhang et al. and Rakib et al., the antiviral activity of selenium ion against SARS-CoV-2 [73] can be enhanced by the combination of Se^−^ with aromatic compounds [74]. Natural HSs samples also have anti-coronaviral activity [75]. Moreover, humic substances interact with Zinc(2+) ions [76] and Selenium(−) ion [77] and stabilize them in chelate form. Apart from increasing their bioavailability, these chelates increase antiviral effects of single ingredients [78]. Similarly, Vitamin C also has antiviral activity against SARS-CoV-2 [79]. In a field study it was reported that humic acid and Vitamin C applied to the drinking water of a broiler chicken during live attenuated virus vaccination, significantly enhanced the effectiveness of immunization against chicken Infectious Bursal Disease virus [80]. This is probably a result of the synergistic effects of HAs and Vitamin C on the host organism.

This proof-of-concept study shows that the Test Article, a combination of peat extract HS and Zn^2+^/Se^−^ based nutritional supplements, possesses measurable, dose-dependent and robust in vitro inhibitory effects on SARS-CoV-2 infection and replication. When molar masses of humic acid are measured normalized by AI, a hundred femtomolar range is apparent, whereas for Selenium ion, a picomolar range for FA, the inhibition constants are in the decimal picomolar range, and for Ascorbic Acid and Zinc ions they are in the hundred-picomolar range. Our in vitro study did not aim to decipher the antiviral actions of each active ingredient in the investigated product, so further experimental research is to be conducted on Selenium, Vitamin C separately, and especially with regard to HSs combinations. 

The exact molecular mechanism of the antiviral effect of HSs is not yet clear. A recent study supposes that natural HSs samples as polyelectrolytes can block the interaction between coronaviral spike glycoproteins and cell membrane receptors [57]. In presenting indirect, host-related antiviral action, HSs can directly suppress oxidative stress, by recombination with intermediate free radicals. HSs can bind to proteins and these protein-HSs aggregates influence various coagulation factors [75]. HSs have been also shown to protect cells against viral infection, e.g., by reducing the expression of tumour necrosis factor alpha, cyclooxygenase2, and prostaglandin E2 secretion in human monocyte cultures, while increasing the resistance of cell membranes to polarization effects [75]. Selenium ions reduce the oxidative stress during viral infection [81]. Zinc ions could both protect cell membranes against viral infection and reduce the replication of viruses by directly blocking key viral enzymes [82]. Vitamin C has also shown some direct virucidal effects against SARS-CoV-2 in vitro, but its molecular mechanism is not yet clear [79].

Furthermore, we postulate a synergistic effect of these ingredients on the cellular homeostatic and antioxidant mechanisms. Ascorbic acid and humic acids were proven to be highly synergistic antioxidants below 0.01 M of ascorbic acid concentration [70]. They form a complex that can also bind Zn^2+^ ions. By increasing cellular antioxidant protection turnover using Se^−^ for glutathione peroxidase, and zinc ions that are also necessary for this enzyme, indirect host-based processes are also strengthened beside the known direct antiviral actions of HSs and Zn^2+^ ions. As we tested a multi-ingredient mixture, it is currently unknown which components have the largest contribution to the measured effect. The main direct antiviral/replication inhibitory effects are probably exerted by FA and Zn^2+^ ions. The hypothesis of this proof-of-concept study should be further tested in pre-clinical animal models of peroral administration, and in clinical trials. These in vitro results are by no means a direct sign of human clinical applicability with a therapeutic intent, but they do provide direction for further investigations on the effects of materials possessing proven in vitro antiviral activity used in combination. We postulate that peroral, safe, and effective nutritional interventions can be designed to prevent severe COVID-19. This is a process starting with in vitro effect studies, moving to peroral investigations using in vivo (e.g., humanized mice or hamster) COVID-19 challenge models, and leading to different designs of clinical nutritional studies. The results presented here should show that humic substances in synergistic combinations of ingredients with known antiviral effects should be a primary subject of such a development process.

The picomolar concentration ranges also indicate that further in vivo studies should be conducted. These values are at least one magnitude lower than the in vitro inhibition values of almost all other available antiviral molecules. 

## 5. Conclusions

Antiviral activity of a humic substance containing ascorbic acid, Se^−^ and Zn^2+^ ions intended as a nutritional supplement material was investigated against SARS-CoV-2 virus B1.1.7 Variant of Concern (“Alpha Variant”) in a VeroE6 cell line. Results show that this combination has a significant in vitro antiviral effect at a very low concentration range of its intended active ingredients. Even picomolar concentration ranges of humic substances, vitamin C and Zn^2+^/Se^−^ ions, in the given composition were enough to achieve 50% viral replication inhibition in the applied SARS-CoV-2 virus inhibition test. This antiviral effect can be caused by the components’ additive effects on a cell-virus system (e.g., antioxidant effect, reduction in viral replication, enhancement of the resistance of cell membrane, reduction in inflammatory mediator cytokine secretion, etc.) in a synergistic manner. Further, in vivo challenge experiments and clinical intervention studies are warranted using appropriately designed humic substance containing candidate formulations.

## Figures and Tables

**Table 1:** Ingredients of the tested “ZnSeC-Humicin” powder nutritional supplement material.

Component	Concentration	Performance Characteristics	
		Lower Limit of Measurement	Uncertainty of Measurement, R%
Humidity	8.5 m/m%	0.01 m/m%	±10.0
Vitamin C	39.6 g/100 g	0.0001 g/100 g	±10.0
Humic acid	0.5 m/m%	0.1 m/m%	±10.0
Fulvic acid	65.9 m/m%	0.1 m/m%	±10.0
Zn^2+^	60,900 mg/kg	0.5 mg/kg	±10.0
Se^−^	23.2 mg/kg	0.01 mg/kg	±10.0

**Table 2:** Characteristics of the inhibition curve of the Test Article against SARS-CoV-2 Alpha VOC.

Parameter	Fit Value	Confidence Interval at 95%
M	7.23 × 10^4^	(51.52 × 10^4^, 94.95 × 10^4^)
a	1739.98	(621.80, 2858.00)
b	4.84 × 10^−3^	(−14.12 × 10^−3^, 23.80 × 10^−3^)

**Table 3:** Calculated API quantities for the observed ID50 effect 1740-fold dilution, measured in the applied 200 microliter volumes.

Active Ingredient in the Test Material (AI) Name	AI Quantity in the Test Material Solution mg	Molar Mass, Da	Calculated Molar Concentration Per Well (as Test Material Ingredient) at ID_50_, pM
Fulvic Acids, Peat Origin	0.1137	2310	70.8
Ascorbic Acid	0.0747	176.12	610
Humic Acids, Peat Origin	0.0009	3160–26,400	0.049–0.409
Zinc (++) ions	0.0114	65.38	251
Selenium (−) ions	0.0004	78.97	7.28

## Data Availability

The data presented in this study are available on request from the corresponding author.

## References

[1] Erdtman H.G.H. (1933). Studies on the formation of complex oxidation and condensation products of phenols. A contribution to the investigation of the origin and nature of humic acid. Part I.—Studies of the reactivity of simple monocyclic quinones. Proc. R. Soc. London. Ser. A Contain. Pap. A Math. Phys. Character.

[2] Waksman S.A. (1936). Húmus Origin, Chemical Composition, and Importance in Nature.

[3] Stevenson F.J. (1994). Humus Chemistry: Genesis, Composition, Reactions.

[4] Steinberg C.E.W., Meinelt T., Timofeyev M.A., Bittner M., Menzel R. (2008). Humic substances. Environ. Sci. Pollut. Res..

[5] Schnitzer M. (1978). Humic Substances: Chemistry and Reactions. Soil Organic Matter.

[6] Nieder R., Benbi D.K., Reichl F.X. (2018). Soil Components and Human Health.

[7] Lag J., Hadas A., Fairbridge R.W., Muñoz J.C.N., Pombal X.P., Cortizas A.M., Almendros G., Chesworth W. (2008). Humic Substances. Encyclopedia of Soil Science.

[8] Alvarez-Puebla R.A., Valenzuela-Calahorro C., Garrido J.J. (2006). Theoretical study on fulvic acid structure, conformation and aggregation. A molecular modelling approach. Sci. Total Environ..

[9] Alvarez-Puebla A.R., Garrido J.J., Aroca R.F. (2004). Surface-Enhanced Vibrational Microspectroscopy of Fulvic Acid Micelles. Anal. Chem..

[10] Lieke T., Steinberg C.E.W., Pan B., Perminova I.V., Meinelt T., Knopf K., Kloas W. (2021). Phenol-rich fulvic acid as a water additive enhances growth, reduces stress, and stimulates the immune system of fish in aquaculture. Sci. Rep..

[11] Chon K., Cho J., Shon H.K. (2013). Advanced characterization of algogenic organic matter, bacterial organic matter, humic acids and fulvic acids. Water Sci. Technol..

[12] Chin Y.-P., Aiken G., O’Loughlin E. (1994). Molecular weight, polydispersity, and spectroscopic properties of aquatic humic substances. Environ. Sci. Technol..

[13] Asakawa D., Kiyota T., Yanagi Y., Fujitake N. (2008). Optimization of Conditions for High-Performance Size-Exclusion Chromatography of Different Soil Humic Acids. Anal. Sci..

[14] Schellekens J., Buurman P., Kalbitz K., Zomeren A.V., Vidal-Torrado P., Cerli C., Comans R.N.J. (2017). Molecular Features of Humic Acids and Fulvic Acids from Contrasting Environments. Environ. Sci. Technol..

[15] Sutton R., Sposito G. (2005). Molecular structure in soil humic substances: The new view. Environ. Sci. Technol..

[16] Nebbioso A., Piccolo A. (2013). Molecular characterization of dissolved organic matter (DOM): A critical review. Anal. Bioanal. Chem..

[17] Ghosal S., Lal J., Singh S.K., Goel R.K., Jaiswal A.K., Bhattacharya S.K. (1991). The need for formulation of Shilajit by its isolated active constituents. Phytother. Res..

[18] Ghosal S., Baumik S., Chattopadhyay S. (1995). Shilajit induced morphometric and functional changes in mouse peritoneal macrophages. Phytother. Res..

[19] Pant K., Singh B., Thakur N. (2012). Shilajit: A Humic Matter Panacea for Cancer. Int. J. Toxicol. Pharmacol. Res..

[20] Mishra T., Dhaliwal H.S., Singh K., Singh N. (2019). Shilajit (Mumie): Current Status of Biochemical, Therapeutic and Clinical Advances. Curr. Nutr. Food Sci..

[21] Kumar Gautam R., Navaratna D., Muthukumaran S., Singh A., Islamuddin, More N. (2021). Humic Substances: Its Toxicology, Chemistry and Biology Associated with Soil, Plants and Environment.

[22] Béres T., Kabdebó S., Ferenc K., Nemeséri L., Szélsy A., Visy L. (1957). Studies on therapeutic application of fulvic acids, with special regard to their liver protecting function. Magy Állatorv. Lapja.

[23] Béres T., Király I., Bóna E., Lővei E., Róbert S. (1958). Tőzeg-fulvósavval szerzett therapiás tapasztalataink. Orvosi Hetilap.

[24] Klöcking R., Björn H. (2005). Medical aspects and applications of humic substances. Biopolymers for Medical and Pharmaceutical Applications.

[25] De Melo B.A., Motta F.L., Santana M.H.A. (2016). Humic acids: Structural properties and multiple functionalities for novel technological developments. Mater. Sci. Eng. C Mater. Biol. Appl..

[26] Murbach T.S., Glavits R., Endres J.R., Clewell A.E., Hirka G., Vertesi A., Beres E., Pasics Szakonyine I. (2020). A toxicological evaluation of a fulvic and humic acids preparation. Toxicol. Rep..

[27] Mosa A., Taha A., Elsaeid M. (2020). Agro-environmental applications of humic substances: A critical review. Egypt. J. Soil Sci..

[28] Szabo J., Vucskits A.V., Berta E., Andrasofszky E., Bersenyi A., Hullar I. (2017). Effect of fulvic and humic acids on iron and manganese homeostasis in rats. Acta Vet. Hung..

[29] Molnar D. (2021). The beneficial effects of humic acid on gastric ulcers in pigs. Int. Pig Top..

[30] Li Y.-M., Li B.-C., Li P., Liu J.-Z., Cui J.-L., Mei Z.-Q. (2011). Effects of Na-FA on gastrointestinal movement and gastric ulcer in mice. J. Chin. Med. Mater..

[31] Kunavue N., Lien T.F. (2012). Effects of Fulvic Acid and Probiotic on Growth Prerformance, Nutrient Digestibility, Blood Parameters and immunity of Pigs. J. Anim. Sci. Adv..

[32] Hullar I., Vucskits A.V., Berta E., Andrasofszky E., Bersenyi A., Szabo J. (2018). Effect of fulvic and humic acids on copper and zinc homeostasis in rats. Acta Vet. Hung..

[33] Kim M.J., Rehman S.U., Amin F.U., Kim M.O. (2017). Enhanced neuroprotection of anthocyanin-loaded PEG-gold nanoparticles against Abeta1–42-induced neuroinflammation and neurodegeneration via the NF-KB /JNK/GSK3beta signaling pathway. Nanomedicine.

[34] Cornejo A., Jiménez J.M., Caballero L., Melo F., Maccioni R.B. (2011). Fulvic acid inhibits aggregation and promotes disassembly of tau fibrils associated with Alzheimer’s disease. J. Alzheimer’s Dis..

[35] Gnananath K., Nataraj K.S., Rao B.G., Kumar K.P., Mahnashi M.H., Anwer M.K., Umar A., Iqbal Z., Mirza M.A. (2020). Exploration of fulvic acid as a functional excipient in line with the regulatory requirement. Environ. Res..

[36] Jacob K.K., Prashob P.K.J., Chandramohanakumar N. (2019). Humic Substances as a Potent Biomaterials for Therapeutic and Drug Delivery System—A Review. Int. J. Appl. Pharm..

[37] Schepetkin I., Khlebnikov A., Kwon B.S. (2002). Medical drugs from humus matter: Focus on mumie. Drug Dev. Res..

[38] Vetvicka V., Vashishta A., Fuentes M., Baigorri R., Garcia-Mina J.M., Yvin J.-C. (2013). The Relative Abundance of Oxygen Alkyl-Related Groups in Aliphatic Domains Is Involved in the Main Pharmacological-Pleiotropic Effects of Humic Acids. J. Med. Food.

[39] Winkler J., Ghosh S. (2018). Therapeutic Potential of Fulvic Acid in Chronic Inflammatory Diseases and Diabetes. J. Diabetes Res..

[40] Vucskits A., Hullár I., Bersenyi A., Andrásofszky E., Tuboly T., Szabó J. (2010). Effect of fulvic acid and humic acid. Part 1. Economic indexes, immunostimulant effect. Magyar Állatorvosok. Lapja.

[41] Vucskits A.V., Hullár I., Bersényi A., Andrásofszky E., Kulcsár M., Szabó J. (2010). Effect of fulvic and humic acids on performance, immune response and thyroid function in rats. J. Anim. Physiol. Anim. Nutr..

[42] Van Rensburg C.E.J. (2015). The Antiinflammatory Properties of Humic Substances: A Mini Review. Phytother. Res..

[43] Kotwal G.J., Kaczmarek J.N., Leivers S., Ghebremariam Y.T., Kulkarni A.P., Bauer G., De Beer C., Preiser W., Mohamed A.R. (2005). Anti-HIV, anti-poxvirus, and anti-SARS activity of a nontoxic, acidic plant extract from the Trifollium species Secomet-V/anti-vac suggests that it contains a novel broad-spectrum antiviral. Ann. N. Y. Acad. Sci..

[44] Klöcking R., Sprössig M. (1972). Antiviral properties of humic acids. Experientia.

[45] Helbig B., Klöckinq R., Wutzler P. (1997). Anti-herpes simplex virus type 1 activity of humic acid-like polymers and their o-diphenolic starting compounds. Antivir. Chem. Chemother..

[46] Klöcking R., Helbig B., Schötz G., Schacke M., Wutzler P. (2002). Anti-HSV-1 Activity of Synthetic Humic Acid-Like Polymers Derived from p -Diphenolic Starting Compounds. Antivir. Chem. Chemother..

[47] (1998). Humic Acid Inhibition of HSV Infection.

[48] Lu F.J., Tseng S.N., Li M.L., Shih S.R. (2002). In vitro anti-influenza virus activity of synthetic humate analogues derived from protocatechuic acid. Arch. Virol..

[49] Jooné G.K., Dekker J., Jansen Van Rensburg C.E. (2003). Investigation of the Immunostimulatory Properties of Oxihumate. Z. Für Nat. C.

[50] Van Rensburg C.E.J., Dekker J., Weis R., Smith T.L., Janse Van Rensburg E., Schneider J. (2002). Investigation of the Anti-HIV Properties of Oxihumate. Chemotherapy.

[51] Kornilaeva G., Becovich A. (2004). New Humic Acid Derivative as Potent Inhibitor of HIV-1 Replication. Med. Gen. Med..

[52] Orlov A.A., Zherebker A., Eletskaya A.A., Chernikov V.S., Kozlovskaya L.I., Zhernov Y.V., Kostyukevich Y., Palyulin V.A., Nikolaev E.N., Osolodkin D.I. (2019). Examination of molecular space and feasible structures of bioactive components of humic substances by FTICR MS data mining in ChEMBL database. Sci. Rep..

[53] Zhernov Y.V., Konstantinov A.I., Zherebker A., Nikolaev E., Orlov A., Savinykh M.I., Kornilaeva G.V., Karamov E.V., Perminova I.V. (2021). Antiviral activity of natural humic substances and shilajit materials against HIV-1: Relation to structure. Environ. Res..

[54] Kotwal G.J. (2008). Genetic diversity-independent neutralization of pandemic viruses (e.g., HIV), potentially pandemic (e.g., H5N1 strain of influenza) and carcinogenic (e.g., HBV and HCV) viruses and possible agents of bioterrorism (variola) by enveloped virus neutralizing com. Vaccine.

[55] Tiwari S.K., Dicks L.M.T., Popov I.V., Karaseva A., Ermakov A.M., Suvorov A., Tagg J.R., Weeks R., Chikindas M.L. (2020). Probiotics at War Against Viruses: What Is Missing from the Picture?. Front. Microbiol..

[56] Köntös Z. (2021). Efficacy of “Essential Iodine Drops” against Severe Acute Respiratory Syndrome-Coronavirus 2 (SARS-CoV-2). PLoS ONE.

[57] Uspenskaya E.V., Syroeshkin A.V., Pleteneva T.V., Kazimova I.V., Grebennikova T.V., Fedyakina I.T., Lebedeva V.V., Latyshev O.E., Eliseeva O.V., Larichev V.F. (2021). Nanodispersions of Polyelectrolytes Based on Humic Substances: Isolation, Physico-Chemical Characterization and Evaluation of Biological Activity. Pharmaceutics.

[58] Larenas-Linnemann D., Rodriguez-Perez N., Arias-Cruz A., Blandon-Vijil M.V., Del Rio-Navarro B.E., Estrada-Cardona A., Gereda J.E., Luna-Pech J.A., Navarrete-Rodriguez E.M., Onuma-Takane E. (2020). Enhancing innate immunity against virus in times of COVID-19: Trying to untangle facts from fictions. World Allergy Organ. J..

[59] Grant W.B., Lahore H., McDonnell S.L., Baggerly C.A., French C.B., Aliano J.L., Bhattoa H.P. (2020). Evidence that Vitamin D Supplementation Could Reduce Risk of Influenza and COVID-19 Infections and Deaths. Nutrients.

[60] Bae M., Kim H. (2020). The role of vitamin C, vitamin D, and selenium in immune system against COVID-19. Molecules.

[61] He L., Zhao J., Wang L., Liu Q., Fan Y., Li B., Yu Y.-L., Chen C., Li Y.-F. (2021). Using nano-selenium to combat Coronavirus Disease 2019 (COVID-19)?. Nano Today.

[62] Quiles J.L., Rivas-Garcia L., Varela-Lopez A., Llopis J., Battino M., Sanchez-Gonzalez C. (2020). Do nutrients and other bioactive molecules from foods have anything to say in the treatment against COVID-19?. Environ. Res..

[63] Te Velthuis A.J., van den Worm S.H., Sims A.C., Baric R.S., Snijder E.J., van Hemert M.J. (2010). Zn(2+) inhibits coronavirus and arterivirus RNA polymerase activity in vitro and zinc ionophores block the replication of these viruses in cell culture. PLoS Pathog..

[64] Ash M. (2021). Nutritional Support for Immunity Against Viruses Including the Coronavirus. Clinical Educatuion.

[65] Saar R.A., Weber J.H. (1982). Fulvic acid: Modifier of metal-ion chemistry. Environ. Sci. Technol..

[66] Kretzschmar R., Christl I. (2001). Proton and metal cation binding to humic substances in relation to chemical composition and molecular size. Spec. Publ. R. Soc. Chem..

[67] Bertoli A.C., Garcia J.S., Trevisan M.G., Ramalho T.C., Freitas M.P. (2016). Interactions fulvate-metal (Zn(2)(+), Cu(2)(+) and Fe(2)(+)): Theoretical investigation of thermodynamic, structural and spectroscopic properties. Biometals.

[68] Jones M.N., Bryan N.D. (1998). Colloidal properties of humic substances. Adv. Colloid Interface Sci..

[69] Chen L., Liang J. (2020). An overview of functional nanoparticles as novel emerging antiviral therapeutic agents. Mater. Sci. Eng. C Mater. Biol. Appl..

[70] Smirnova O.V., Efimova I.V., Khil’ko S.L. (2012). Antioxidant and pro-oxidant activity of ascorbic and humic acids in radical-chain oxidation processes. Russ. J. Appl. Chem..

[71] Krezel A., Maret W. (2016). The biological inorganic chemistry of zinc ions. Arch. Biochem. Biophys..

[72] Marreiro D.D.N., Cruz K.J.C., Oliveira A.R.S.D., Morais J.B.S., Freitas B.D.J.E.S.D.A., Melo S.R.D.S., Dos Santos L.R., Cardoso B.E.P., Dias T.M.D.S. (2021). Antiviral and immunological activity of zinc and possible role in COVID-19. Br. J. Nutr..

[73] Zhang J., Saad R., Taylor E.W., Rayman M.P. (2020). Selenium and selenoproteins in viral infection with potential relevance to COVID-19. Redox Biol..

[74] Rakib A., Nain Z., Sami S.A., Mahmud S., Islam A., Ahmed S., Siddiqui A.B.F., Babu S.M.O.F., Hossain P., Shahriar A. (2021). A molecular modelling approach for identifying antiviral selenium-containing heterocyclic compounds that inhibit the main protease of SARS-CoV-2: An in silico investigation. Brief. Bioinform..

[75] Hafez M., Popov A.I., Zelenkov V.N., Teplyakova T.V., Rashad M. (2020). Humic substances as an environmental- friendly organic wastes potentially help as natural anti-virus to inhibit COVID-19. Sci. Arch..

[76] Boguta P., Sokołowska Z. (2016). Interactions of Zn(II) Ions with Humic Acids Isolated from Various Type of Soils. Effect of pH, Zn Concentrations and Humic Acids Chemical Properties. PLoS ONE.

[77] Aguilar F., Charrondiere U.R., Dusemund B., Galtier P., Gilbert J., Gott D.M., Grilli S., Guertler R., Kass G.E.N., Koenig J. (2009). Chromium(III)-, iron(II)- and selenium-humic acid/fulvic acid chelate and supplemented humifulvate added for nutritional purposes to food supplements. EFSA J..

[78] Constantinescu-Aruxandei D., Frîncu R., Capră L., Oancea F. (2018). Selenium Analysis and Speciation in Dietary Supplements Based on Next-Generation Selenium Ingredients. Nutrients.

[79] Abobaker A., Alzwi A., Alraied A.H.A. (2020). Overview of the possible role of vitamin C in management of COVID-19. Pharmacol. Rep..

[80] ELSayed E.S.M., AME A., HA S., Deeb K. (2007). The Effect of Humic Acid and Ascorbic Acid on Immunization of Chickens Against Infectious Bursal Disease. Assuit Venetrary Med. J..

[81] Tomo S., Saikiran G., Banerjee M., Paul S. (2021). Selenium to selenoproteins—Role in COVID-19. EXCLI J..

[82] Kumar A., Kubota Y., Chernov M., Kasuya H. (2020). Potential role of zinc supplementation in prophylaxis and treatment of COVID-19. Med. Hypotheses.

